# Reserve Design under Climate Change: From Land Facets Back to Ecosystem Representation

**DOI:** 10.1371/journal.pone.0126918

**Published:** 2015-05-15

**Authors:** Richard R. Schneider, Erin M. Bayne

**Affiliations:** Department of Biological Sciences, University of Alberta, Edmonton, Alberta, Canada; University of New South Wales, AUSTRALIA

## Abstract

Ecosystem distributions are expected to shift as a result of global warming, raising concerns about the long-term utility of reserve systems based on coarse-filter ecosystem representation. We tested the extent to which proportional ecosystem representation targets would be maintained under a changing climate by projecting the distribution of the major ecosystems of Alberta, Canada, into the future using bioclimatic envelope models and then calculating the composition of reserves in successive periods. We used the Marxan conservation planning software to generate the suite of reserve systems for our test, varying the representation target and degree of reserve clumping. Our climate envelope projections for the 2080s indicate that virtually all reserves will, in time, be comprised of different ecosystem types than today. Nevertheless, our proportional targets for ecosystem representation were maintained across all time periods, with only minor exceptions. We hypothesize that this stability in representation arises because ecosystems may be serving as proxies for land facets, the stable abiotic landscape features that delineate major arenas of biological activity. The implication is that accommodating climate change may not require abandoning the conventional ecosystem-based approach to reserve design in favour of a strictly abiotic approach, since the two approaches may be largely synonymous.

## Introduction

A large body of literature on reserve design has been developed over the past few decades, emphasizing a structured systematic approach [1,2]. Coarse-filter applications [3] have involved quantification of existing ecological patterns, analysis of the extent to which ecological features are represented within existing protected areas, and the application of optimization algorithms to efficiently fill gaps in representation [4,5].

Global climate change presents a challenge to conventional reserve design in that ecological elements selected for protection are expected to shift from their current (protected) locations under most climate scenarios [6–8]. Consequently, it has been suggested that the whole approach to representation needs to be reconsidered [9,10]. A proposed alternative focuses on capturing variability in stable abiotic landscape features, referred to as land facets, instead of ecological elements [3,9–13]. The idea underlying this coarse-filter approach is to protect the “arenas” of biological activity, not the temporary occupants of those arenas.

Although the land facet approach has considerable merit, there are several shortcomings related to its practical application. First, the influence of abiotic landscape features such as surficial geology, soils, and topography on ecological patterns is scale dependent [14,15]. In some places, geophysical relationships that contribute to diversity at the local scale may have little connection to the broader ecological patterns that are most relevant to coarse-filter conservation. For example, in the province of Alberta, Canada, surficial geology is almost entirely glacial in origin. Typical features such as glacial lake beds and moraines are present throughout the province in a fine-scale pattern that is unrelated to regional ecological patterns, which are characterized by broad gradients of change [16]. Protecting a representative sample of surficial geologic features would, in this setting, provide little assurance that the major arenas of biological activity have been protected.

Another abiotic feature commonly used to define land facets is soil type. The issue here is that, at the regional scale, soil type is sometimes at the end of a causal chain involving climate and vegetation, not at the front. For example, the spatial distribution of grassland, parkland, and boreal forest ecosystems in western Canada is primarily a function of available moisture [17,18]. Although soil type is demonstrably different among these regions, this is an outcome of climatically-driven vegetation patterns, and associated soil development processes, not a cause [19,20]. It is therefore somewhat misleading to portray land facets based on the broad soil types in this region as abiotic landscape features.

A third problem with a strictly abiotic approach to representation is a lack of practical guidance for how land facets should be created from the available suite of abiotic elements. Which abiotic elements should be included? How does scale affect this decision? How should continuous entities such as elevation and latitude be converted into discrete units? On what basis should selected abiotic elements be combined to create facets? Should all inputs be weighted equally, even if some appear to be redundant? There are no generic answers to these questions. Cluster analysis could be used to objectively define categories, but this provides no assurance that the categories that have been defined are optimal in terms of reflecting present and future patterns of biodiversity. Nor does it provide insight into the appropriate number of categories to use in any given area.

Given the practical shortcomings of a strictly abiotic approach to reserve design, the case for its use in place of the conventional ecosystem-based approach may be less compelling than its proponents have suggested. Conversely, the failings of the conventional approach to reserve design have not been convincingly demonstrated. The assertion that reserve designs based on ecosystem representation are destined to fail under climate change appears to be based only on concerns that ecosystems selected for protection will shift from their current locations as the climate warms [21]. It has not been shown that these changes in ecosystem distribution will result in gaps in representation that threaten the core objectives of coarse-filter conservation over broad spatial extents.

While we support the premise that land facets are the key to stability under climate change [9], we believe that ecosystems could serve as effective proxies for land facets when designing reserves. We reason that if land facets are the arenas for biological diversity then ecological patterns should reflect these facets. The linkage must be bidirectional. Using ecosystems to delineate land facets has the benefit of providing guidance about which landscape features are ecologically important and which are of little relevance to conservation planning. In addition, changes in the composition of biotic communities along abiotic gradients, such as latitude and elevation, provide an indication of where differentiation is reasonable and warranted.

If our reasoning is sound, then reserves selected on the basis of ecosystem representation should continue to protect a representative sample of biodiversity regardless of future changes in climate and associated shifts in ecological patterns. The purpose of this study is to determine the extent to which this is true. Our test is based on projected changes in ecosystem distribution derived from bioclimatic envelope models [22]. We focus on moderate to extreme changes in climate, assuming that if the system is robust under high levels of warming then it will be generally robust.

A positive result will provide planners and conservation managers confidence that reserve designs that have been developed or are being developed on the basis of conventional ecosystem representation are not destined to fail under climate change. This is highly relevant in Alberta, Canada, where conservation planning efforts are currently underway, and more generally to any jurisdiction that has spent considerable time and effort developing ecosystem classifications for the purpose of conservation planning.

## Methods

### Ecosystem Classification

Our study area is the province of Alberta, Canada (662,600 km^2^). A hierarchical system of ecosystem classification, developed by the provincial government, divides the province into six Natural Regions and 21 Natural Subregions [16]. This classification has supplied the provincial ecological context within which resource management activities, including reserve design, have been planned and implemented since the 1970s.

Decisions concerning ecosystem delineation were made on a case-by-case basis by a team of domain experts, drawing on their first-hand knowledge of provincial ecological patterns, augmented by digital spatial data and field visits. The team used a variety of inputs—including vegetation, landform, climate, soils, and hydrology—to arrive at an appropriate boundary for each ecosystem type. Refinement of the boundaries has occurred over the years as additional information has become available. The approach used in Alberta is a derivative of earlier systems of classification developed at the national scale [23] and variations of this approach are used in other Canadian Provinces (e.g., [24]).

The interplay between biotic and abiotic elements was deemed to be critical to defining meaningful ecosystem boundaries [16]. It follows that the Natural Regions classification intrinsically captures some aspects of a land facet approach, though the emphasis on using ecological patterns for guidance sets it apart. Although climate was used as an input to the classification process this was mainly in a secondary role (e.g., for the spatial extrapolation of observed biotic patterns where vegetation inventory data were incomplete).

### Reserve Design

**Fig 1:**
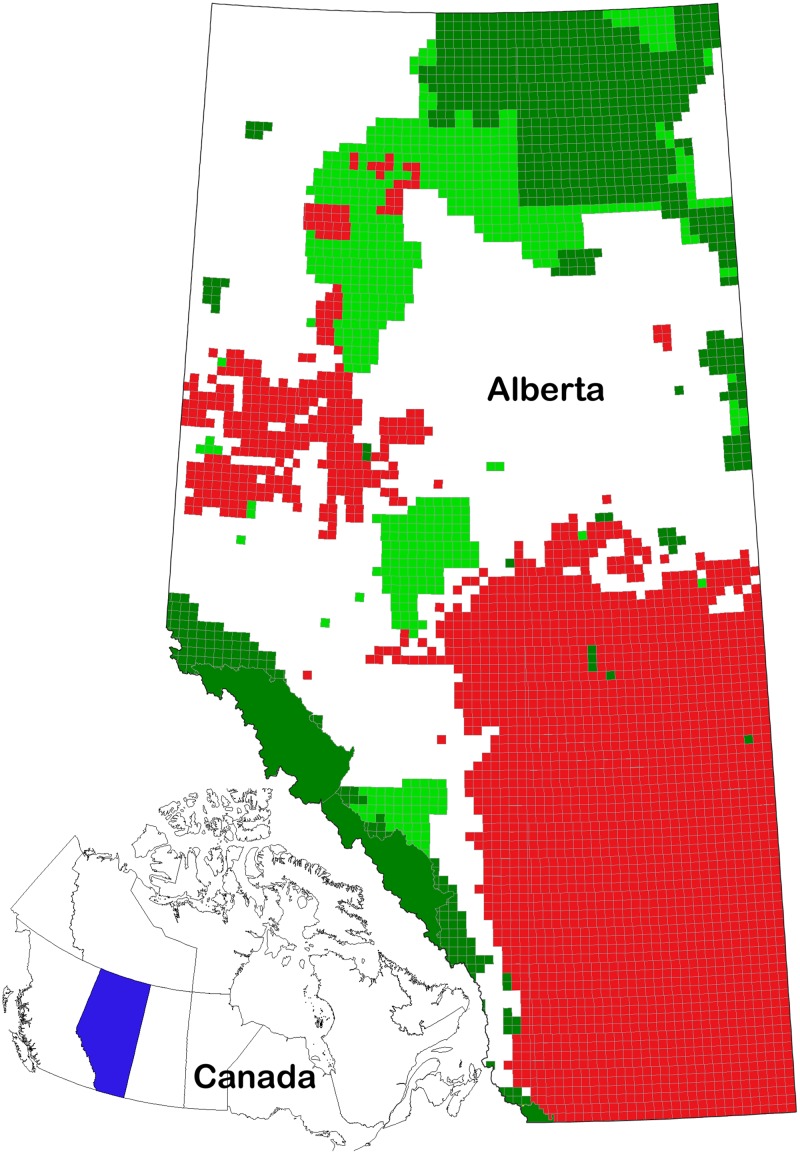
Representative Marxan reserve design. Parameters included a 20% representation target and high boundary penalty. Dark green = existing protected areas; light green = planning units selected by Marxan; red = agricultural exclusion zone. Inset: location of Alberta within Canada.

Protected areas, including sites announced in two recent regional land-use plans, cover 14.4% of Alberta. The distribution of these protected areas is heavily skewed to the Rocky Mountains and the northeast corner of the province; therefore, gaps in representation exist for many Natural Subregions (Fig 1). For our investigation of the effects of climate change on ecosystem representation we wanted to begin with a reserve system that did not have such gaps in representation present at the outset. We also wanted to explore how changes in the level of representation and the degree of contiguity (i.e., reserve clumping) would affect the results. Therefore, rather than using the existing reserve network for our analysis, we used a set of reserve designs generated with the Marxan conservation planning software [25].

Our approach to reserve design was based on methodology developed in an earlier study, where Marxan was used to generate reserve designs that achieved specified coarse-filter representation targets while minimizing economic opportunity costs and maximizing intactness [26]. We limited the Marxan analysis to Alberta’s non-agricultural land-use zone, where coarse-filter conservation planning is currently being undertaken by the Government of Alberta in the context of regional planning. We excluded the less intact southern agricultural zone from the analysis (Fig 1) because habitat protection efforts in this area have generally involved a fine-filter approach, aimed at protecting remnant patches of native grasslands and the habitat of threatened species. Excluding this area also helped make the study more tractable, as the complexities of private land ownership were avoided and we did not need to project the movement of northern United States climate spaces into southern Alberta.

Townships (~9500 ha administrative units; Fig 1) were used as the planning unit (n = 6954) in Marxan. Townships within the provincial protected area network were forced into the design if 50% or more of the township was protected.

We generated reserve designs with representation targets of 15%, 20% and 30%. For example, a representation target of 20% meant that at least 20% of each Natural Subregion had to be represented in the final reserve design. Economic opportunity cost was based on the net present value, at the township scale, of the four main types of resources present in our study area—conventional natural gas, conventional oil, oil sands, and forest products—using models developed by Hauer et al. [27]. We incorporated intactness on the basis of the density of linear features, summarized by township. Linear features were derived from the Alberta Base Features dataset and included roads, pipelines, and seismic lines. Economic opportunity cost and intactness did not have explicit targets, but instead, the model treated both variables as (equally weighted) costs that it sought to minimize as it worked to achieve the specified representation targets.

Marxan includes a penalty factor that can be applied to the total length of reserve boundaries. As the boundary length penalty is increased, contiguous planning units are increasingly favoured, resulting in clumping of reserves and an increase in their mean size. We used two settings for the boundary penalty: none, and a high value selected to maximize the clumping of reserves.

Each of the six design scenarios (two levels of boundary penalty times three levels of representation target) was run 500 times in Marxan to obtain the “best” design possible given the various constraints imposed. Although the optimization algorithm used by Marxan cannot guarantee that the optimal design has actually been identified, in our earlier study we found that additional runs did not result in meaningful improvements in the design [26]. In any case, it is the differences among the six design scenarios that are of interest to this study, not minute variations within individual scenarios.

### Bioclimatic Envelope Modeling

Our approach to bioclimatic envelope modelling was based on methodology developed by Hamann et al. [28] and extended in subsequent studies [7,29,30]. In this approach, statistical models are used to define the unique climate space, or “envelope”, of individual ecosystems, based on current eco-climatic associations. Once developed, these models can be used to predict ecosystem type using the present or future climate as an input. When applied to future climates, the working assumption is that existing eco-climatic associations will remain intact (to a point), and so a shift in regional climate envelope will result in a spatial shift in regional ecosystem distribution, albeit with a lag.

We obtained the climate data for this study, including mean annual temperature, mean annual precipitation, mean warmest monthly temperature, mean coldest monthly temperature, seasonality, growing degree-days above 5°C, and a measure of available moisture, from the ClimateWNA model [31]. In addition to providing high-resolution historical climate data for the 1961–1990 reference period, ClimateWNA provides downscaled projections for 24 General Circulation Models (GCMs) used in the Intergovernmental Panel on Climate Change Fourth Assessment [32]. For our analysis of future conditions we used projections from two GCMs: the ECHAM5 model, selected to represent a median warming scenario (relative to other models) and the HADGEM model, selected to represent an extreme warming scenario. The A2 emission scenario was used for both models [32].

**Fig 2:**
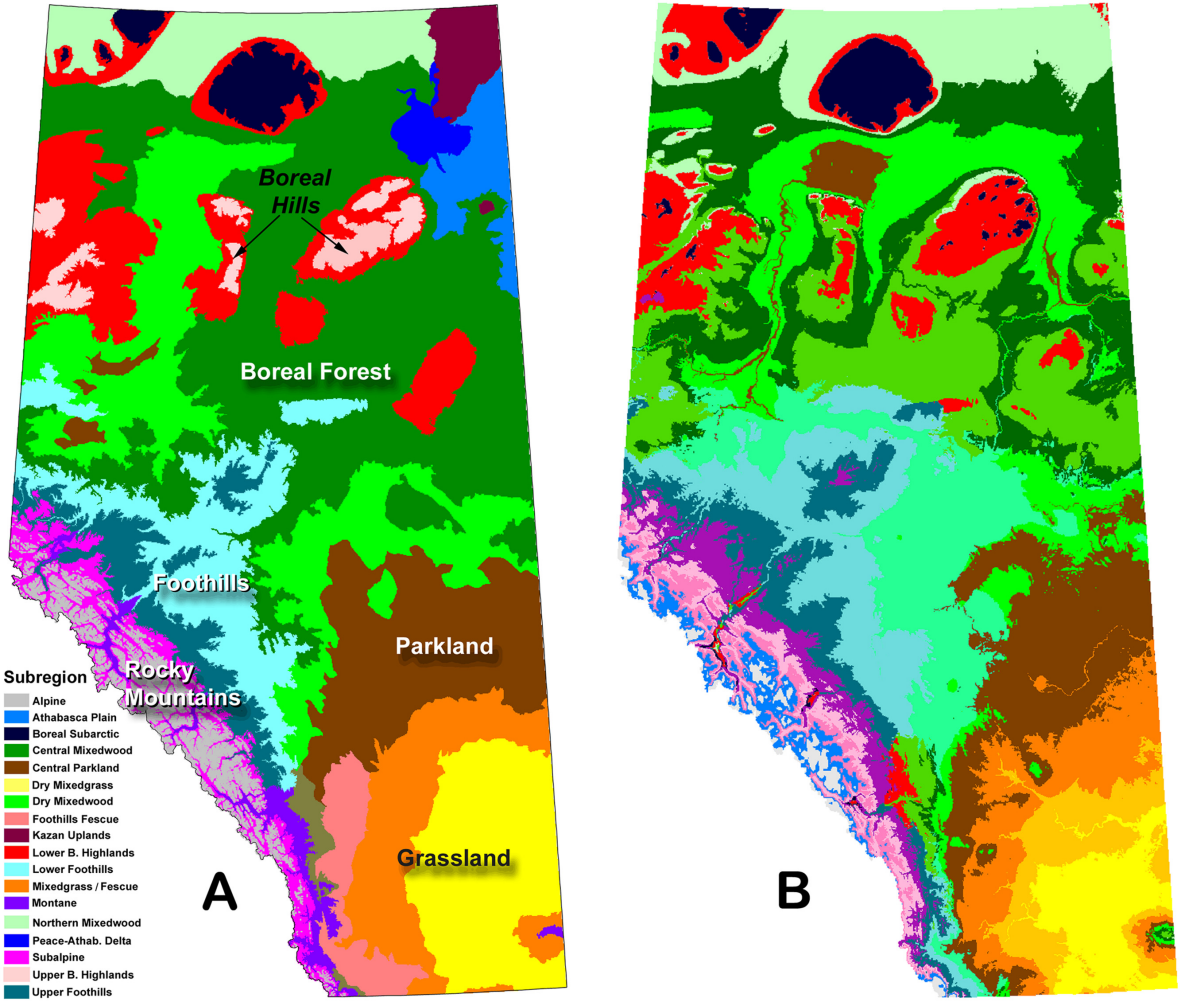
Comparison of classification systems. Panel A = Natural Subregions of Alberta. Panel B = Cluster analysis of ClimateWNA historical climate variables with k = 18 clusters.

We used Natural Subregions as the ecosystem variable in our analysis (Fig 2a). We eliminated three Subregions in northeast Alberta—Kazan Upland, Athabasca Plain, and Peace-Athabasca Delta—because they were defined primarily on the basis of unique soil or hydrological conditions [16]. The dominant influence of site conditions within these Subregions precludes the effective use of bioclimatic envelope models; however, for the same reason we expect they will continue to support distinct ecosystems regardless of future changes in climate. For the rest of the province, a k-means cluster analysis using the ClimateWNA historical climate data illustrates the overriding influence of climate on ecological patterns, supporting our use of bioclimatic envelope models (compare Fig 2a and 2b).

A variety of approaches can be used to create the statistical linkage between ecosystem type and climate. We used different approaches in different parts of the province in an attempt to maximize the robustness and explanatory power of the models. Where Subregions constitute an ordered set responding to a relatively uniform climatic gradient we used ordinal regression, implemented using the lrm regression procedure in the R programming language [33], to build the model (S1 Note). This applied to a latitudinal gradient involving Grasslands, Parkland, and Boreal Forest Plain Subregions [16,17] and also to an elevational gradient involving Subregions comprising the boreal hill systems (Fig 2a).

The situation in the Foothills and Rocky Mountain Regions is more complex because the lower elevation slopes abut multiple climatic zones, meaning that a single climatic gradient could not be assumed. In these regions we used the Random Forests package, implemented in the R programming language, to build the bioclimatic models. Random Forests uses a computer learning approach to construct a classification tree, with no a priori assumptions concerning the structure of the input data [34]. Additional detail concerning the bioclimatic envelope models is available in Schneider [35].

We generated maps of projected ecosystem distribution at future points in time by using our bioclimatic models in combination with climate data from the ECHAM5–A2 and the HADGEM—A2 GCMs. Projections were made for three time periods, reflecting the temporal resolution of the GCMs: 2011–2040, 2041–2070, and 2071–2100 (hereinafter referred to as 2020s, 2050s, and 2080s). For our analysis of ecosystem representation over time we used the Raster package, implemented in the R programming language [33], to calculate the percent of each ecosystem type that occurred within the reserve network, repeating this process for each combination of reserve design and ecosystem distribution map. The resolution of the analysis was 1 km^2^.

## Results

Fig 1 provides an example of the reserve networks generated by Marxan, in this case with a 20% representation target and high boundary penalty. The other reserve networks are provided as S1–S5 Figs. Because very little of the Dry Mixedwood, Lower Foothills, and Upper Foothills are currently protected (1.4%, 0.6%, and 2.5%, respectively), these Subregions were consistently emphasized in the Marxan designs. The Grassland and Parkland Regions are also poorly represented (~ 1% protection); however, these gaps could not be filled because they occur in the agricultural exclusion zone.

**Fig 3:**
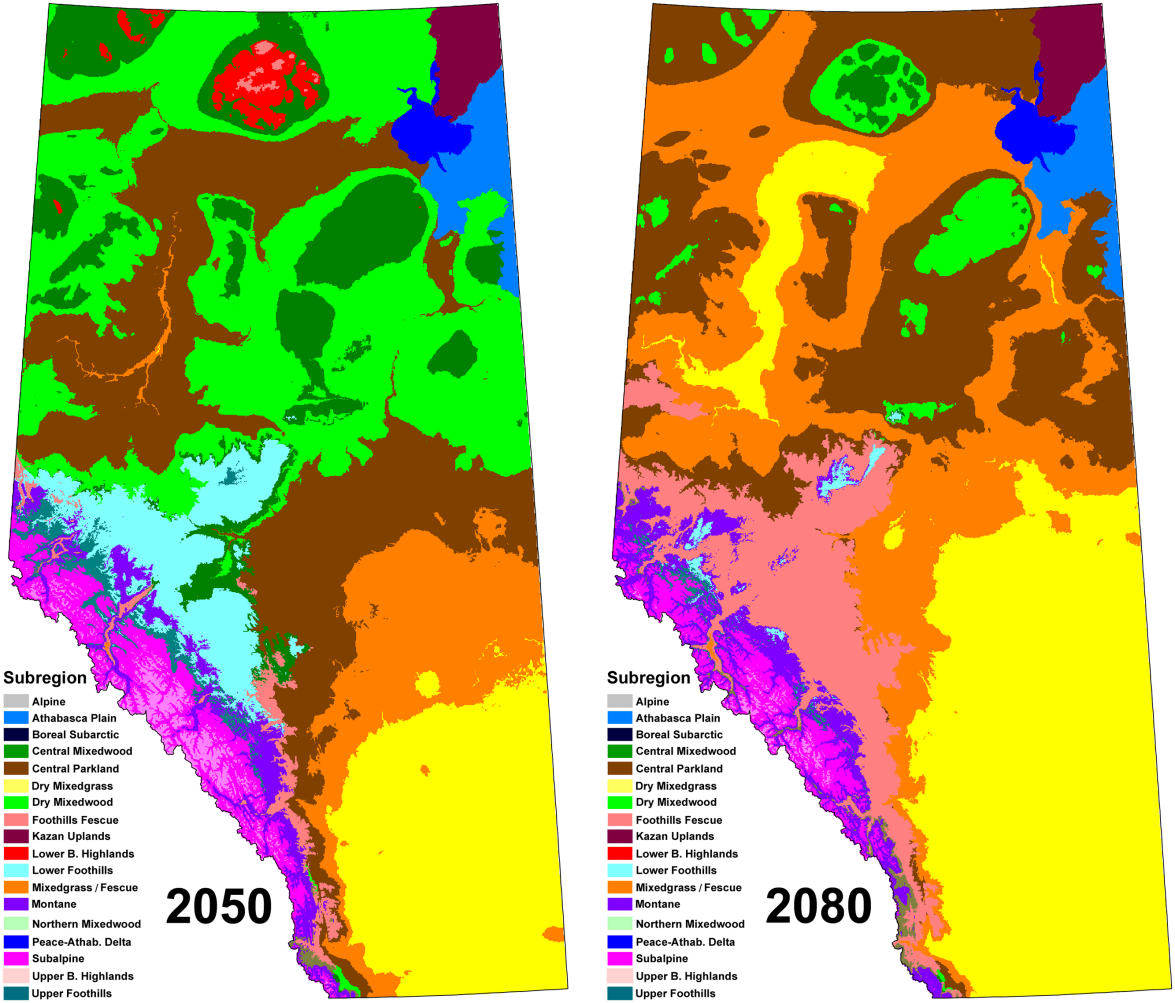
Climate envelope projections for the ECHAM5–A2 GCM (median model). Panel A = 2050s; panel B = the 2080s. Three Subregions in northeast Alberta were not modeled.

The GCMs we examined project a rise in provincial mean annual temperature of 4.3°C and 6.4°C, and a rise in mean annual precipitation of 8.9% and 5.9%, for the ECHAM5-A2 and HADGEM-A2 models, respectively. Under these climate projections, the bioclimatic envelope models predict that the climate currently associated with boreal forest (58% of the province) will be restricted to the tops of the boreal hill system by the end of the century (Fig 3 and S6 Fig). The boreal climate space will be replaced by climates currently associated with Parkland and Grassland ecosystems. In the Foothills and Rocky Mountains the climate envelopes will generally shift upslope, with a mixture of climatic influences at the lowest elevations.

Ecosystem representation was generally maintained or increased over time relative to what the Marxan designs achieved with current ecosystem distributions (Table 1; S1 and S2 Data). The only exception to this outcome was a small number of systems in 2020 that did not achieve their representation target when it was set at 30% (though they were still close). The achievement of representation targets in the Grassland and Parkland Region improved over time, as these Regions shifted into protected areas in northern parts of the province.

**Table 1:** Number of occurrences, across all 12 GCM and design scenarios, that ecosystem representation targets were not achieved, by Subregion and time period.

Natural Region	Natural Subregion	Today	2020	2050	2080
Boreal	Central Mixedwood	0	3	0	0
	Northern Mixedwood	0	0	0	0
	Lower Boreal Highlands	0	0	0	0
	Upper Boreal Highlands	0	0	0	0
	SubArctic	0	0	0	0
	Dry Mixedwood	0	0	0	0
Foothills	Lower Foothills	0	0	0	0
	Upper Foothills	0	0	0	0
Grassland	Dry Grassland	12	12	12	12
	Mixed Grassland	12	12	6	6
	Foothills Fescue	12	12	7	2
Parkland	Central Parkland	12	12	1	1
	Foothills Parkland	12	12	6	0
Rocky Mountain	Alpine	0	0	0	0
	Montane	0	0	0	0
	SubAlpine	0	0	0	0

## Discussion

Our results suggest that, in Alberta, a reserve system designed on the basis of coarse-filter ecosystem representation will continue to achieve proportional representation targets as the climate warms in future decades. We hypothesize that this outcome is a consequence of the linkages that exist between ecosystem distribution, regional climatic patterns, and (in turn) static landscape features, including major landforms and latitudinal gradients [16]. Because of these linkages, a reserve system designed to achieve full ecosystem representation concomitantly achieves effective representation of ecologically-relevant abiotic landscape features, and thus indirectly benefits from the stability that a land facet approach provides [9,11].

Our findings are subject to various forms of uncertainty. As with any statistical approach, the reliability of predictions is highest when they do not require extrapolation beyond the originating dataset. In the case of bioclimatic envelope models, this means reliability is highest when future climate envelopes are represented somewhere within the study area as it currently exists. In a previous study we determined that this should be the case for most of the province, except the southern prairies, under low to moderate levels of warming [35]. Climate spaces that currently exist within the province are generally expected to shift northwards or upslope. Under these conditions, exemplified by the median (ECHAM5) warming scenario, we can expect that ecosystems should track the changes in climate as we have projected, at least at the coarse scale of ecosystem definition used in this study. Eco-climatic linkages are very strong at the regional scale (Fig 2), and given that surficial geology is mostly glacial in origin, there is little to prevent the spatial substitution of ecosystem types. This is what appears to have occurred during the Hypsithermal period (~6,000 BP) when temperatures temporarily warmed by approximately 2°C [18].

Predicting ecosystem responses under higher levels of warming, represented by the HADGEM GCM in this study, is more problematic. Novel climates are more likely to manifest, and the rapid rate of change may disrupt existing eco-climatic associations [36]. Thus, while our findings indicate that representational stability can be expected under all levels of warming, the level of uncertainty is much higher under high levels of warming.

Other forms of potential uncertainty related to modeling exist, but are of less concern. Ecosystems novel to Alberta will arise in the southern prairies, as the climate space from northern U.S. shifts into Alberta [36]. But this region was not included in our analysis of reserve representation (partly for this reason), and so transitions within it did not affect our results. More generally, a shortcoming of bioclimatic envelope models is that they do not take ecological lags into account [22,37]. This has no bearing on our study because our interest is in the eventual state of the system under the projected future climate, and not the timing of the changes. Finally, the choice of representation target or reserve design had no appreciable effect on the outcome.

Our finding of a positive outcome with respect to the maintenance of ecosystem representation rests on the assumption that species will be able to track their preferred climatic and ecological conditions as the climate warms. But many species may have difficulty doing so at the rate necessary, particularly those with a low intrinsic rate of dispersal and those with diminished vitality and resilience as a result of anthropogenic disturbance [38,39]. Barriers to movement, including regions where habitat quality has been compromised (e.g., agricultural lands) are another factor that may hamper the ability of species to shift their range [40]. These issues have management implications, which are discussed below.

A final issue is the extent to which our findings are applicable to other regions. In particular, whereas ecosystem distribution is primarily influenced by climate in most of our study area, abiotic landscape features may be the dominant influence in other areas [11]. This is the case in the three Subregions in northeast Alberta that we excluded from our analysis [16]. We do not view this as a serious concern because the tighter the linkage between abiotic landscape features and ecosystem type, the more that ecosystems become synonymous with land facets. The question of which approach to use as the basis for representation—ecosystems or land facets—becomes increasingly moot in these cases, as they amount to the same thing.

### Management Implications

Our findings suggest that the application of a strictly abiotic land facet approach may not be necessary for accommodating climate change. The conventional approach to reserve selection based on coarse-filter ecosystem representation should remain a viable option, particularly for jurisdictions that have already developed ecosystem classifications. Furthermore, land managers can have confidence that existing sites selected on the basis of ecosystem representation should continue to serve a useful role in coarse-filter biodiversity conservation.

In areas where ecosystem classification is unavailable, an abiotic approach to reserve design still merits consideration because data requirements are less onerous [3]. However, to be reliable, decisions made concerning the choice of abiotic elements and how they are combined into land facets need to be subjected to some sort of field validation to ensure that the designated land facets are ecologically relevant and protect desired biodiversity elements [9]. The benefit of the ecosystem approach is that the ecological relevance of the classification is established at the outset.

It should be noted that our prediction of stability applies only to the achievement of proportional representation targets. If the GCMs used in our analysis are indicative of the future climate, then individual reserves will eventually be comprised of completely different ecosystem types than today. Although this represents a major departure from conventional expectations of stability within reserves, it does not preclude achievement of the objectives of coarse-filter conservation. This is because the coarse-filter approach is meant to protect biodiversity in a broad sense, without reference to the location of individual species. The same cannot be said of fine-filter conservation. The role of static reserves for the protection of individual species will need to be reexamined in light of anticipated shifts in the ranges of focal species.

The absolute area of several ecosystem types, especially those in the Boreal Region, is expected to decline—in some cases to zero. This presents a new dimension to conservation planning. The prospect of a marked decline of large ecosystems is discomforting, even if proportional representation is maintained within reserves. And it seems to demand a response. For example, managers may seek to increase the level of protection of climatically-threatened ecosystems through the protection of climatic refugia [41,42].

Conversely, it can be argued that historical status has no particular relevance in a world characterized by progressive climatic change [43–46]. Under this view, coarse-filter conservation would remain focused on providing protection from anthropogenic disturbance for the benefit of biodiversity in general, whatever its composition may be. This implies a conceptual shift in the conservation baseline, from a static historical state (seek to maintain what we had, where we had it), to a dynamic state (seek to maintain what we would have, at any given time, in the absence of agricultural clearing, forest harvesting, and other anthropogenic disturbances). A societal dialog will be needed to fully explore these options and ultimately provide guidance to land managers and policy makers.

In addition to value-based questions concerning conservation objectives, there are also practical issues inherent in responding to anticipated ecosystem declines through the establishment of climatic refugia. First, the establishment of additional reserves to serve as climatic refugia could come at the cost of not meeting conventional representation targets, given that the overall amount of land available for protection is usually limited [5]. This opportunity cost needs to be considered. Second, as the ecosystems in question decline in extent, they are also moving in space. Microrefugia may persist, but large static in-place refugia do not seem like a viable option in Alberta, for the moderate to high levels of warming we examined.

Conceptually, the climate refuge concept could be applied within a dynamic framework. However, the practical challenges involved in continuously protecting a minimal viable area of a moving target would be formidable. That said, there may be cases where opportunities for establishing refugia are fairly obvious. For example, based on regional topography, climate envelope projections [35], and evidence from the Hypsithermal period [18], it seems clear that the boreal hill system will serve as the final refuge of boreal forest in Alberta. This information could be used to inform a conventional reserve design.

It is worth noting that declines in one ecosystem type will be offset by increases in another. In Alberta, this includes ecosystem types poorly represented in the current reserve system, such as Parkland and Grassland. These ecosystems are expected to gain increased protection as they move from private agricultural land in the south to large protected areas that exist on public lands in the north. Ensuring that these potential conservation gains are realized is another issue that conservation managers will need to devote attention to.

Facilitating species movement will be a critical component of climate change adaptation, to ensure that species are able to track their preferred environmental conditions and make full use of the reserve system [10,47]. Steps that can be taken at the design stage include orienting reserves along climatic gradients and identifying sites that can serve as stepping stones between existing reserves [48,49]. Management of the matrix will also be important, both in terms of reducing barriers to movement and to buy time for species that are unable to respond quickly enough [50]. For example, sites that are likely to remain stable the longest (e.g., micro-refugia) could be identified and assigned an extra level of protection in land management plans [51,52]. Assisted migration is another management option, though the costs involved and risks of unintended consequences may limit the extent of its application [53,54].

In conclusion, our findings suggest that the conventional approach to reserve design should remain viable for coarse-filter applications in the face of climate change. However, a conceptual shift is needed, in that ecosystems are not being identified and protected for their own sake, but as proxies for land facets which provide the basis for long-term stability. In addition, climate change adds new dimensions to the conservation planning process and further increases the importance of facilitating species movement among reserves.

## Supporting Information

S1 Data Representation achieved by Subregion type and time period for each reserve design under the ECHAM5 GCM. (XLSX)

S2 Data Representation achieved by Subregion type and time period for each reserve design under the HADGEM GCM. (XLSX)

S1 Fig Representative Marxan reserve design. Parameters included a 20% representation target and no boundary penalty.
(TIF)

S2 Fig Representative Marxan reserve design. Parameters included a 15% representation target and no boundary penalty.
(TIF)

S3 Fig Representative Marxan reserve design. Parameters included a 15% representation target and high boundary penalty.
(TIF)

S4 Fig Representative Marxan reserve design. Parameters included a 30% representation target and no boundary penalty.
(TIF)

S5 Fig Representative Marxan reserve design. Parameters included a 30% representation target and high boundary penalty.
(TIF)

S6 Fig Climate envelope projections for the HADGEM—A2 GCM (extreme model). Panel A = 2050s; panel B = the 2080s. Three Subregions in northeast Alberta were not modeled.
(TIF)

S1 Note Model construction and fit for the bioclimatic envelope models. (PDF)
