# Supplementary figures and images for: Reserve Design under Climate Change: From Land Facets Back to Ecosystem Representation
Source: PLoS One. 2015 May 15;10(5):e0126918. doi: 10.1371/journal.pone.0126918 (PMC4433178; doi:10.1371/journal.pone.0126918)

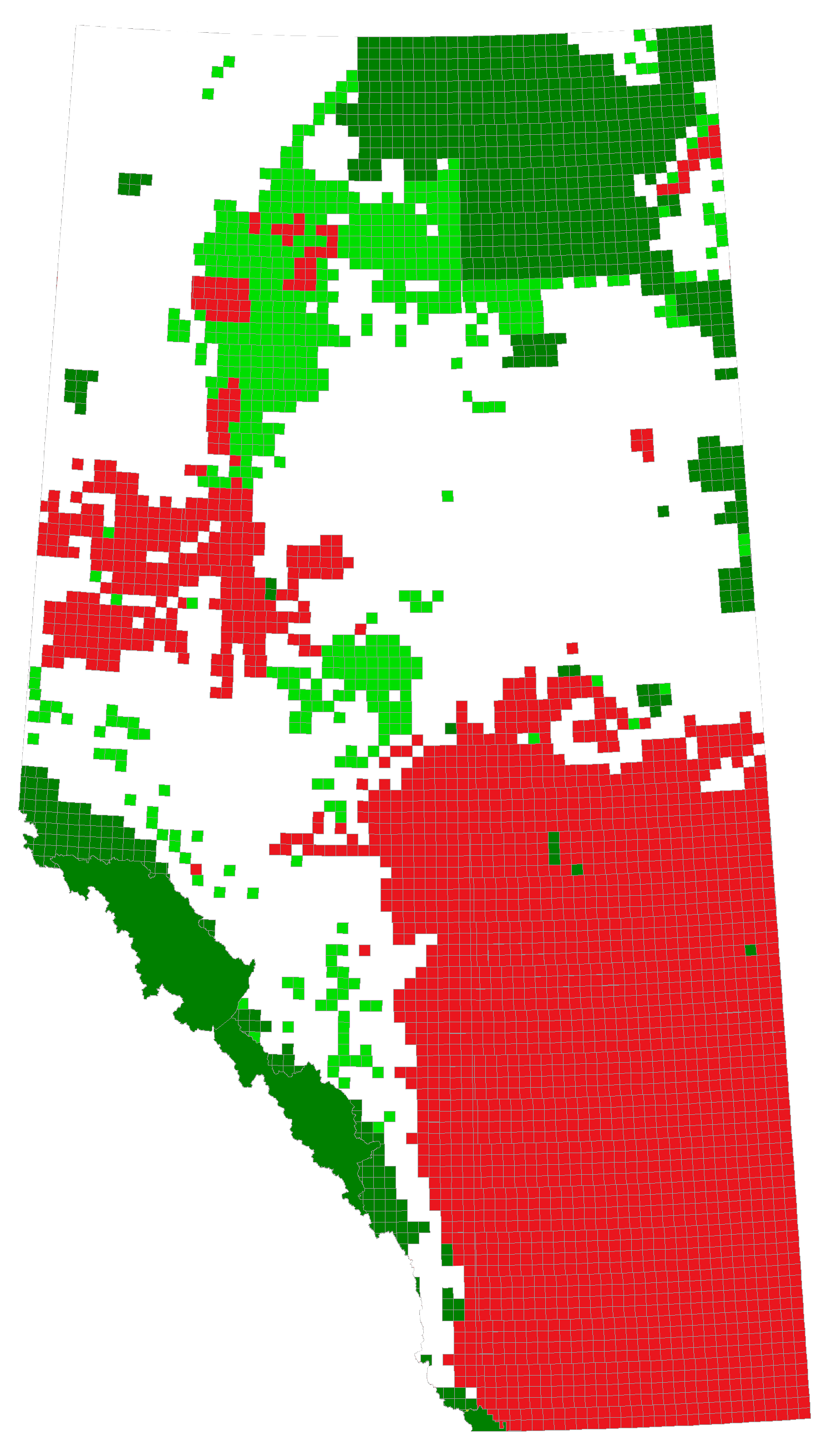

Supplement: S1 Fig — Parameters included a 20% representation target and no boundary penalty. (TIF) [file pone.0126918.s003.tif]

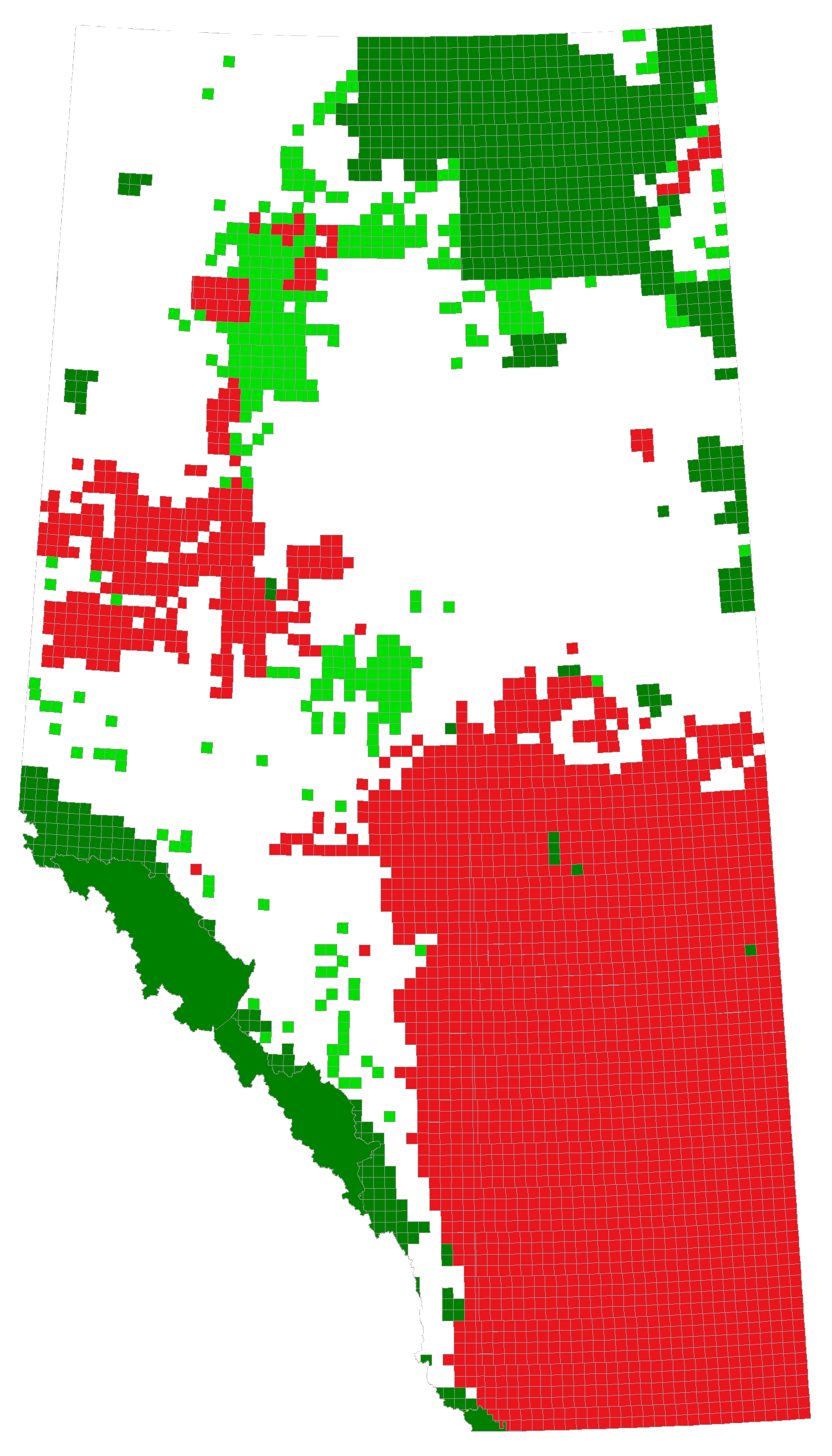

Supplement: S2 Fig — Parameters included a 15% representation target and no boundary penalty. (TIF) [file pone.0126918.s004.tif]

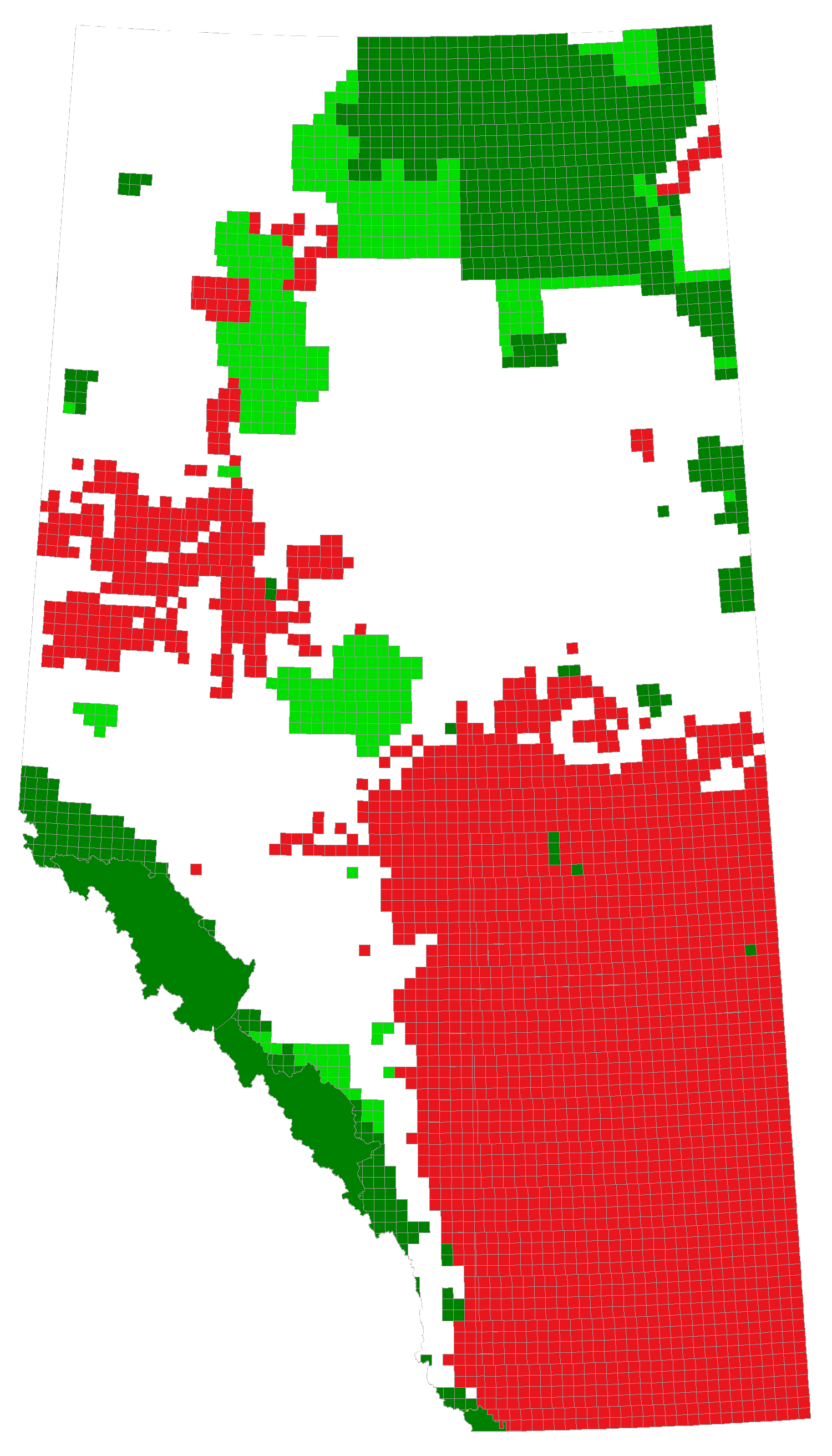

Supplement: S3 Fig — Parameters included a 15% representation target and high boundary penalty. (TIF) [file pone.0126918.s005.tif]

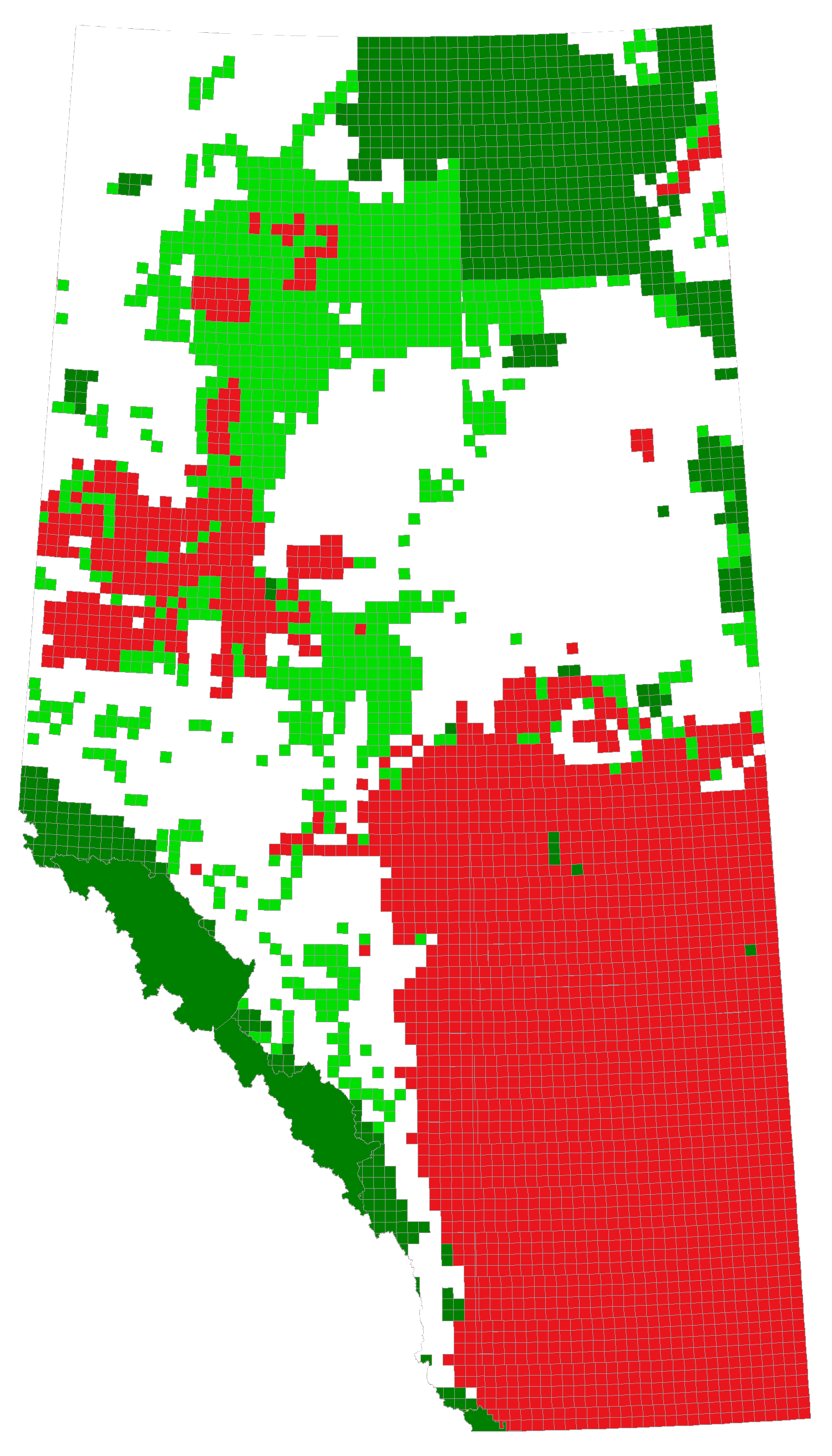

Supplement: S4 Fig — Parameters included a 30% representation target and no boundary penalty. (TIF) [file pone.0126918.s006.tif]

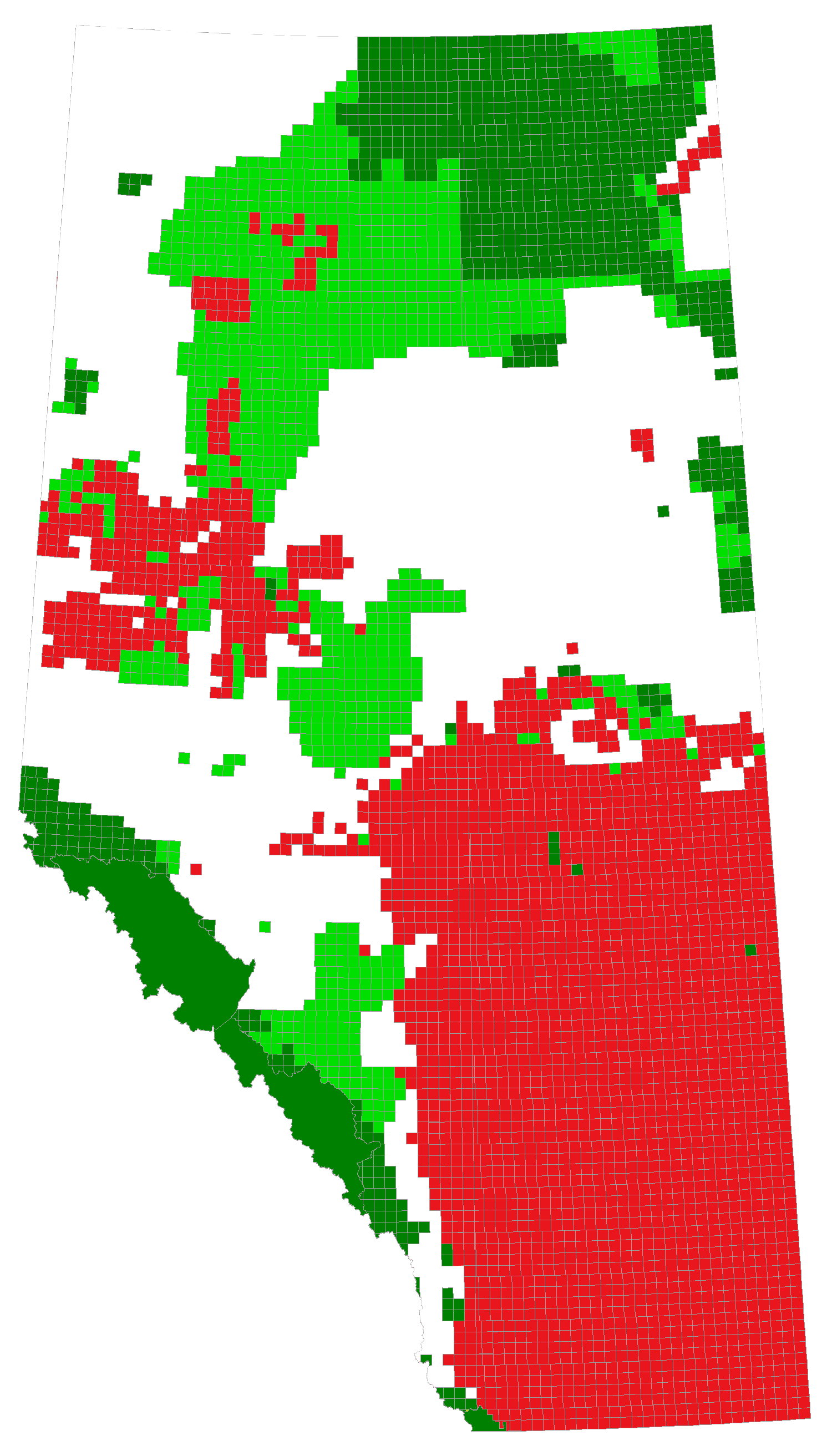

Supplement: S5 Fig — Parameters included a 30% representation target and high boundary penalty. (TIF) [file pone.0126918.s007.tif]

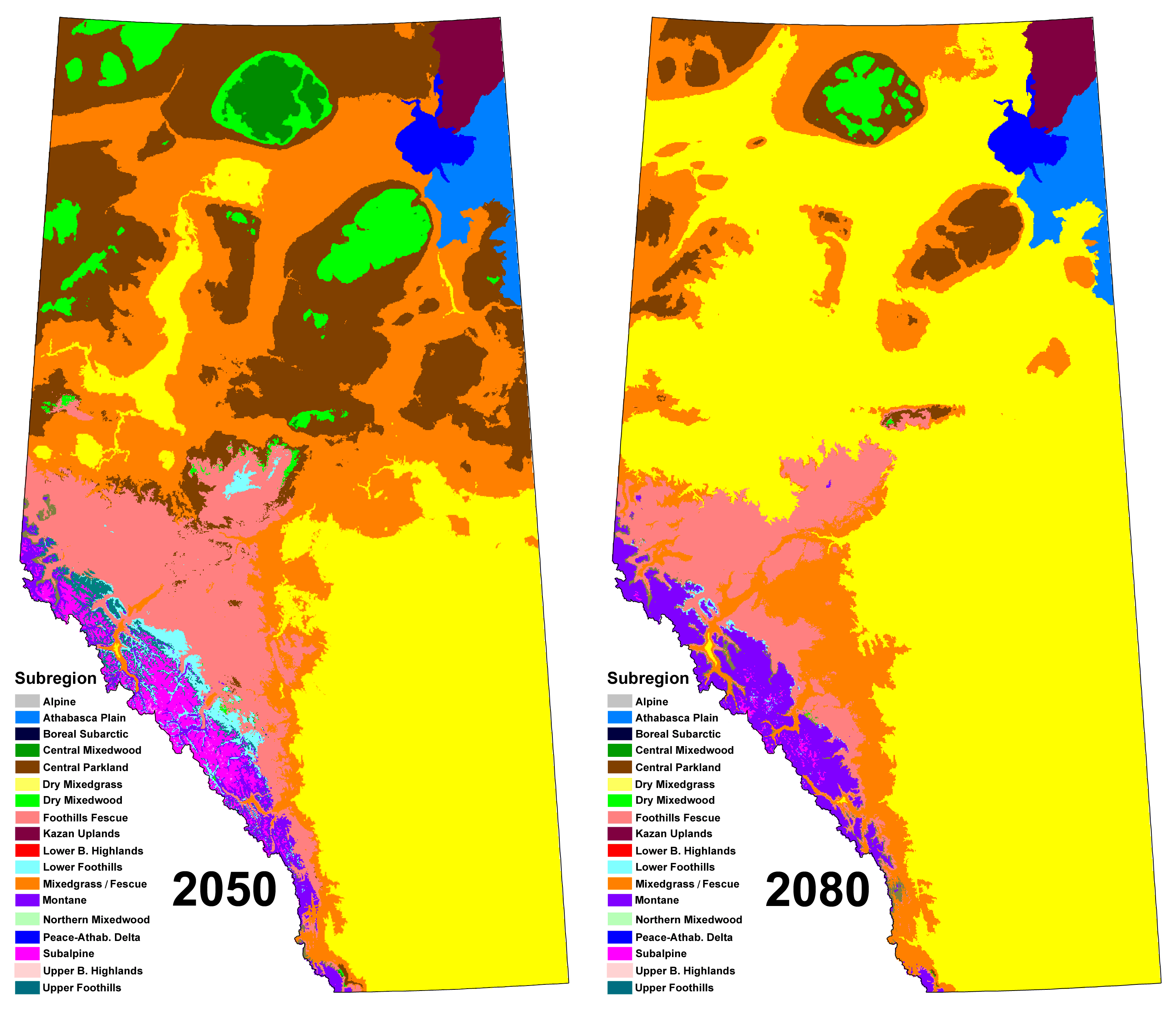

Supplement: S6 Fig — Panel A = 2050s; panel B = the 2080s. Three Subregions in northeast Alberta were not modeled. (TIF) [file pone.0126918.s008.tif]
