# Supplementary material for: Reserve Design under Climate Change: From Land Facets Back to Ecosystem Representation
Source: PLoS One. 2015 May 15;10(5):e0126918. doi: 10.1371/journal.pone.0126918 (PMC4433178; doi:10.1371/journal.pone.0126918)
Supplement: S1 Note — (PDF) [file pone.0126918.s009.pdf]

S1 Note. Output summary for the bioclimatic envelope models.

Output summary for the Grassland to Boreal Model.

n = 60,000
Model: lrm(formula = NSR ~ (CMI + MAT + TD)^2 + MAP + GDD5, data = nsr.samp)
Model likelihood ratio test: p <0.0001
r^2 = 0.95

Variable	Coefficient	S.E.	p
y>=2	-25.7036	0.8314	<0.0001
y>=3	-34.2469	0.8406	<0.0001
y>=4	-40.1881	0.8455	<0.0001
y>=5	-46.339	0.8587	<0.0001
CMI	-0.9031	0.0371	<0.0001
MAT	2.9495	0.2047	<0.0001
TD	1.9715	0.0462	<0.0001
MAP	0.0412	0.0014	<0.0001
GDD5	-0.0325	0.0009	<0.0001
CMI * MAT	-0.0502	0.0022	<0.0001
CMI * TD	0.042	0.001	<0.0001
MAT * TD	-0.0252	0.0034	<0.0001

Output summary for the Boreal Highlands Model.

n = 60,000

Model: lrm(formula = NSR ~ (CMI + MAT + TD)^2 + MAP + GDD5, data = nsr.samp)

Model likelihood ratio test: p <0.0001

$r^2 = 0.89$

Variable	Coefficient	S.E.	p
y>=2	45.587	0.8374	<0.0001
y>=3	41.8629	0.8296	<0.0001
y>=4	37.7087	0.8271	<0.0001
CMI	-1.1759	0.0482	<0.0001
MAT	-9.3214	0.2236	<0.0001
TD	-0.858	0.035	<0.0001
MAP	0.0388	0.0011	<0.0001
GDD5	-0.0273	0.0009	<0.0001
CMI * MAT	-0.078	0.0033	<0.0001
CMI * TD	0.021	0.0014	<0.0001
MAT * TD	0.2083	0.0042	<0.0001

Output summary for the Foothills Model

Model: RandomForest(formula = NSR ~ CMI + MAT + MAP + MWMT + MCMT + TD + GDD5 + slope + Eref + AHM + SHM + NFFD + MSP + PAS + EMT + bFFP + FFP + eFFP, data = nsr.samp, mtry = 3, ntree = 500, proximity = FALSE, importance = TRUE)

OOB estimate of error rate: 5.17%
